# Tetherin does not significantly restrict dendritic cell-mediated HIV-1 transmission and its expression is upregulated by newly synthesized HIV-1 Nef

**DOI:** 10.1186/1742-4690-8-26

**Published:** 2011-04-19

**Authors:** Christopher M Coleman, Paul Spearman, Li Wu

**Affiliations:** 1 Center for Retrovirus Research, Department of Veterinary Biosciences, The Ohio State University, Columbus, OH 43210, USA; 2 Departments of Pediatrics and Microbiology and Immunology, Emory University, Atlanta, GA 30322, USA

## Abstract

**Background:**

Dendritic cells (DCs) are among the first cells to encounter HIV-1 and play important roles in viral transmission and pathogenesis. Immature DCs allow productive HIV-1 replication and long-term viral dissemination. The pro-inflammatory factor lipopolysaccharide (LPS) induces DC maturation and enhances the efficiency of DC-mediated HIV-1 transmission. Type I interferon (IFN) partially inhibits HIV-1 replication and cell-cell transmission in CD4^+ ^T cells and macrophages. Tetherin is a type I IFN-inducible restriction factor that blocks HIV-1 release and modulates CD4^+ ^T cell-mediated cell-to-cell transmission of HIV-1. However, the role of type I IFN and tetherin in HIV-1 infection of DCs and DC-mediated viral transmission remains unknown.

**Results:**

We demonstrated that IFN-alpha (IFNα)-induced mature DCs restricted HIV-1 replication and *trans*-infection of CD4^+ ^T cells. Tetherin expression in monocyte-derived immature DCs was undetectable or very low. High levels of tetherin were transiently expressed in LPS- and IFNα-induced mature DCs, while HIV-1 localized into distinct patches in these DCs. Knockdown of induced tetherin in LPS- or IFNα-matured DCs modestly enhanced HIV-1 transmission to CD4^+ ^T cells, but had no significant effect on wild-type HIV-1 replication in mature DCs. Intriguingly, we found that HIV-1 replication in immature DCs induced significant tetherin expression in a Nef-dependent manner.

**Conclusions:**

The restriction of HIV-1 replication and transmission in IFNα-induced mature DCs indicates a potent anti-HIV-1 response; however, high levels of tetherin induced in mature DCs cannot significantly restrict wild-type HIV-1 release and DC-mediated HIV-1 transmission. Nef-dependent tetherin induction in HIV-1-infected immature DCs suggests an innate immune response of DCs to HIV-1 infection.

## Background

Dendritic cells (DCs) are professional antigen presenting cells that bridge innate and adaptive immunity. DCs play an important role in innate immune recognition and activation during HIV infection [1,2]. HIV-1 hijacks DCs to promote viral infection and dissemination [2,3]. Immature dendritic cells (iDCs) in the mucosa are one of the first cells that encounter HIV-1 during initial infection [4,5]. Immature DCs allow productive HIV-1 replication and long-term viral dissemination [6-8]. Depending on the stimulus, maturation of DCs has differential effects on HIV-1 replication and cell-to-cell transmission to CD4^+ ^T cells [6,9-13]. DC-mediated dissemination of HIV-1 occurs through the dissociable processes of *trans*- and *cis*-infection, depending on whether productive viral infection is initiated in DCs [6]. Productive HIV-1 infection of DCs can induce DC maturation and trigger antiviral innate immunity through type I IFN responses [14].

The major DC subtypes include myeloid DCs and plasmacytoid DCs (pDC) [2,3]. pDCs produce type I IFN upon sensing HIV-1 RNA and envelope protein through Toll-like receptor 7 and other intracellular sensors [15,16]. Type I IFNs are antiviral cytokines produced as part of the innate immune response to an infection to limit virus dissemination and regulate adaptive immune responses to clear the virus and protect against re-infection [17]. As a type I IFN, IFNα can inhibit HIV-1 replication in CD4^+ ^T cells and macrophages *in vitro *[18,19]. A recent study indicated that IFNα partially inhibits the cell-to-cell transmission of HIV-1 between CD4^+ ^T cells [20]. However, it is unknown whether IFNα can block HIV-1 replication in DCs or DC-mediated cell-to-cell transmission of HIV-1.

Type I IFNs can induce the expression of HIV-1 restriction factors [21], in particular, APOBEC3 family proteins [22-24], Trim5α [25] and tetherin (BST-2 or CD317) [26,27]. Tetherin is a host transmembrane protein [26,27] and is expressed by a wide-range of human and animal cells [28,29]. Mouse and human pDCs [30,31] and human monocyte-derived DCs (MDDCs) [29] express endogenous tetherin, though its function is not fully understood. Tetherin has been suggested as a component of the innate immune responses [32]. It has been shown that human pDCs express an orphan receptor called immunoglobulin-like transcript 7 (ILT7), which binds to tetherin and down-regulates the IFN responses of pDCs [31]. This study suggested that type I IFN produced by pDCs during viral infection may stimulate neighboring cells to express tetherin, which interacts with ILT7 on pDCs to down-modulate IFN and cytokine responses.

Tetherin restricts release of various enveloped viruses, including a number of retroviruses and several viral proteins function as antagonists of tetherin (reviewed in [32-36]). Tetherin acts as an HIV-1 restriction factor by directly tethering HIV-1 virions to the surface of an HIV-producing cell [27,37], but its effect on incoming HIV-1 virions during cell-to-cell transmission has not been documented. The HIV-1 protein Vpu antagonizes tetherin by causing the degradation [38-41] and the sequestration of tetherin into a perinuclear compartment away from the site of virus assembly [42]. Moreover, Nef and envelope proteins from some simian immunodeficiency viruses (SIV) [43-46] and HIV-2 envelope proteins [42,47] function as antagonists of tetherin in a species-specific manner.

It is unknown whether tetherin plays a role in DC-mediated HIV-1 infection and transmission. Recent studies suggest different roles of tetherin in the cell-to-cell transmission of HIV-1 mediated by CD4^+ ^T cells [48-50]. Casartelli *et al*. reported that tetherin impairs cell-to-cell transmission of HIV-1 in several cell lines and primary CD4^+ ^T cells, and transmission of Vpu-defective HIV-1 to target CD4^+ ^T cells is less efficient than that of wild-type (WT) HIV-1 [49]. By contrast, Jolly *et al. *suggested that tetherin can enhance HIV-1 cell-to-cell transmission, and Vpu-defective HIV-1 is disseminated more efficiently compared with WT HIV-1 in CD4^+ ^Jurkat T cells [48]. Using tetherin-inducible Sup-T1 cells, Kuhl *et al. *recently reported that tetherin expressed on target cells promotes HIV-1 cell-to-cell transfer, while tetherin expressed on donor cells inhibits viral transmission [50]. The discrepancy between these studies may be due to cell-type-dependent variation in tetherin expression levels [49,50], which remains to be confirmed using other primary HIV-1 target cells, such as DCs or macrophages.

In this study, we investigated the role of IFNα and tetherin in MDDC-mediated HIV-1 infection and transmission. We demonstrated that IFNα treatment of DCs restricted DC-mediated HIV-1 infection and transmission to CD4^+ ^T cells. We observed that tetherin expression was transiently upregulated in LPS- or IFNα-matured DCs and knockdown of induced tetherin modestly enhanced mature DC-mediated HIV-1 transmission, but had no significant effect on WT HIV-1 replication in mature DCs. Intriguingly, we found that tetherin was induced by HIV-1 infection of iDCs in a Nef-dependent manner, suggesting that tetherin upregulation is an innate immune response of DCs to HIV-1 infection.

## Results

### IFNα induces DC maturation but does not alter the expression level of HIV-1 receptors

**Figure 1:**
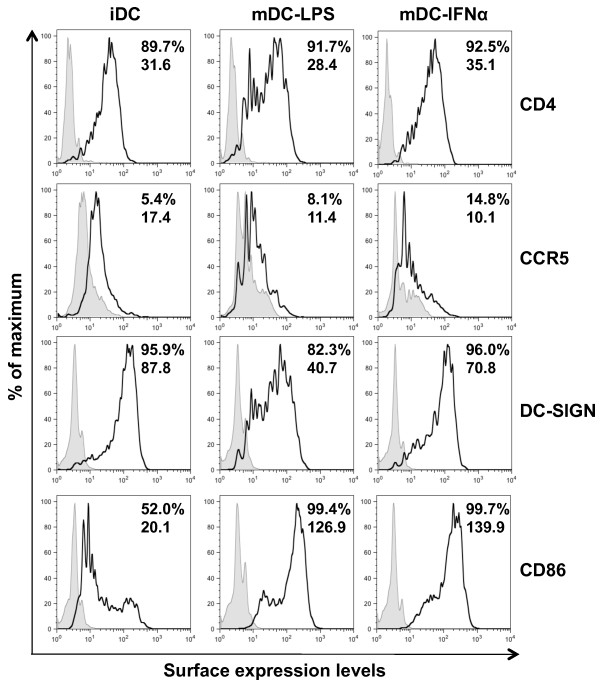
**IFNα induces DC maturation but does not alter the expression of HIV-1 receptors**. iDC, mDC-LPS and mDC-IFNα were stained for cell surface expression of CD4, CCR5, DC-SIGN and CD86. On each histogram, the filled peaks are the controls of isotype or secondary antibody alone and the black peaks represent the staining of specific markers. Top and bottom numbers shown in plots are % positive and the geometric mean values of fluorescence intensity, respectively. Results shown are from DCs from a single donor representative of two independent experiments on DCs from different donors.

To examine the role of type I IFN in DC-mediated HIV-1 infection and transmission, human monocyte-derived iDCs were activated with IFNα to generate mature DCs (mDC-IFNα) and LPS-induced mature DCs (mDC-LPS) were used as positive controls. DCs were separately stained for surface CD86 as a marker of maturation [6,11,14,51], for the HIV-1 receptors CD4 and CCR5, and for the HIV-1 attachment factor DC-SIGN (DC-specific intercellular adhesion molecule-3 grabbing non-integrin). Maturation of DCs with either LPS or IFNα caused significant upregulation of CD86 expression on the cell surface by 6- to 7-fold (Figure 1), indicating that both mature DC types developed a mature DC phenotype. Compared with iDCs, mDC-IFNα did not show any significant differences in the expression of CD4, CCR5 or DC-SIGN, while mDC-LPS showed decreased levels of both CD4 and DC-SIGN (Figure 1). Surface CCR5 was equally expressed at low levels on all DC types (Figure 1). Thus, IFNα-induced maturation of DCs does not significantly affect the expression of HIV-1 receptors.

### IFNα-induced mature DCs do not mediate efficient HIV-1 transmission to CD4^+ ^T cells

**Figure 2:**
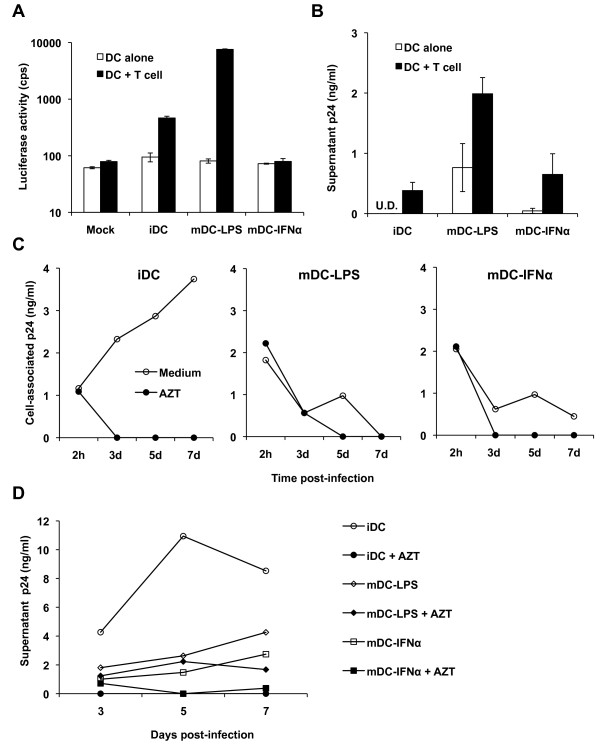
**Transmission and replication of HIV-1 is restricted in mDC-IFNα**. Transmission of HIV-1 by DCs was assessed by incubating DCs with either the single-cycle luciferase reporter HIV-1 or replication-competent HIV-1 NL(AD8) for 2 h, then co-cultured with Hut/CCR5 target cells for 3 or 2 days, respectively; transmission was assessed by whole-cell luciferase assay or release of p24 in supernatants. (A) mDC-IFNα do not enhance transmission of the single-cycle luciferase reporter virus to CD4^+ ^T cells over iDC transmission levels. cps, counts per second. Mock, mock infected iDCs. Data represent mean ± SEM of three independent experiments performed on DCs from three different donors. U.D., undetectable (lower than detection limit). (B) mDC-IFNα do not enhance transmission of HIV-1 NL(AD8) to CD4^+ ^T cells at 2 dpi (days post-infection) relative to iDC transmission levels. Graph represents mean data ± SEM from three independent experiments performed with DCs from three different donors. DCs were infected with WT NL(AD8) and p24 production in the cell lysates (C) or supernatants (D) was monitored after 2 h or 3-7 dpi using a p24 ELISA. AZT was used to assess productive HIV-1 infection. Data are from one experiment and representative of at least two independent experiments.

To assess the effect of IFNα on DC-mediated transmission of HIV-1 to CD4^+ ^T cells, HIV-1-pulsed mDC-IFNα were co-cultured with Hut/CCR5 cells in viral transmission assays. Single-cycle, R5-tropic luciferase reporter HIV-1 was used and viral transmission was determined by measuring luciferase activity in cell lysates of co-cultures [52]. HIV-1-pulsed DCs alone were used as a control for background replication. mDC-LPS showed a 16-fold increase in viral transmission compared with iDC-mediated moderate transmission of HIV-1 to CD4^+ ^T cells (Figure 2A). By contrast, mDC-IFNα failed to enhance single-cycle HIV-1 transmission to CD4^+ ^T cells (Figure 2A).

It has been established that there are two distinct phases in DC-mediated HIV-1 transfer to CD4^+ ^T cells [8]. In the first phase (within 24 hr after infection), incoming HIV-1 is transferred, whereas in the second phase (24-72 hr after infection), newly synthesized HIV-1 can be transmitted [8]. To examine the two-phase HIV-1 transfer, DC-mediated transmission of replication-competent R5-tropic HIV-1 NL(AD8) was assessed by p24 release in supernatants from the co-cultures of HIV-1-pulsed DCs and Hut/CCR5 cells 2 days later. Compared with iDC-mediated HIV-1 transmission, mDC-LPS transmitted HIV-1 to CD4^+ ^T cells 5-fold more efficiently, while mDC-IFNα transmitted HIV-1 only 2-fold more efficiently (Figure 2B). Together, these data indicate that mDC-IFNα do not mediate efficient HIV-1 transmission to CD4^+ ^T cells.

### Productive HIV-1 replication is restricted in IFNα-induced mature DCs

To understand the mechanism by which IFNα treatment restricts DC-mediated HIV-1 transmission, the kinetics of HIV-1 uptake, degradation and replication in mDC-IFNα were assessed. The reverse transcriptase inhibitor azidothymidine (AZT) was used to confirm productive HIV-1 replication in DCs. HIV-1 enters DCs mainly through endocytosis, but productive HIV-1 infection of DCs is dependent upon fusion-mediated viral entry [6,53], therefore, cell-associated p24 can be indicative of either HIV-1 entry pathway in DCs and supernatant p24 represents productive viral replication and/or viral release.

After 2 h incubation of DCs with HIV-1 NL(AD8), cells were washed extensively, aliquoted and cultured for up to 7 days. The amount of HIV-1 uptake by DCs was quantified by measuring the cell-associated p24 at 2 h post-infection. Compared with iDCs, mDC-LPS and mDC-IFNα captured 2-fold more HIV-1 (Figure 2C). Over the time course, iDCs showed increases of both cell-associated p24 (Figure 2C) and released virus (Figure 2D), which were efficiently blocked by AZT, consistent with productive HIV-1 replication. The HIV-1 captured by mDC-LPS were degraded (Figure 2C), or otherwise released into the media over time in a largely replication independent manner (Figure 2D). HIV-1 in mDC-IFNα was rapidly degraded, as the cell-associated p24 reached very low levels at 3 days post-infection (dpi) (Figure 2C). Low levels of HIV-1 release from mDC-IFNα was observed at 5 and 7 dpi, which was significantly reduced in the presence of AZT (Figure 2D), indicating delayed viral replication in mDC-IFNα. These data suggest that IFNα maturation of DCs blocks HIV-1 replication.

### Pro-inflammatory stimuli upregulate tetherin expression in DCs

**Figure 3:**
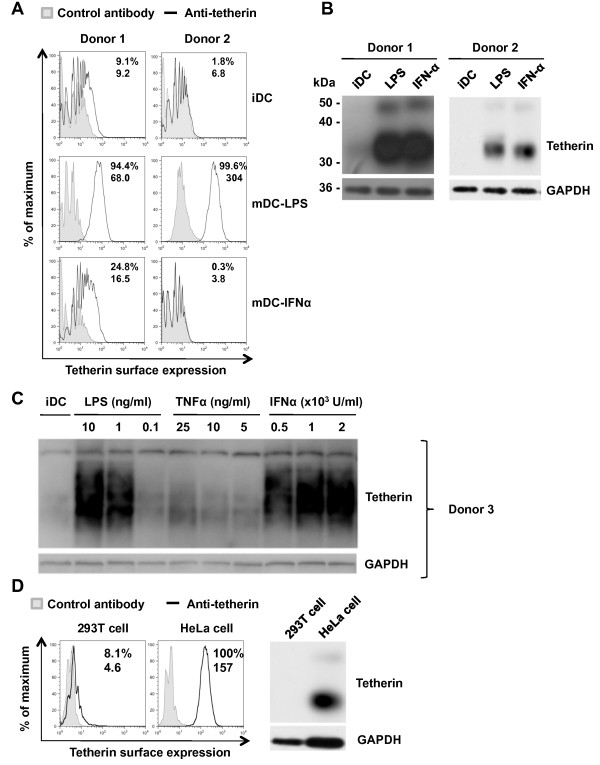
**Pro-inflammatory stimuli upregulate tetherin expression in DCs**. Tetherin expression on iDCs, mDC-LPS and mDC-IFNα from two different donors was assessed by (A) flow cytometry and (B) immunoblotting. (C) TNF-α treatment of DCs modestly upregulates tetherin expression compared with mDC-LPS and mDC-IFNα. Tetherin expression was detected by immunoblotting. (D) HEK293T and HeLa cells were used as negative and positive controls, respectively. Numbers shown in flow cytometry plots are % positive (top) and the geometric mean values of fluorescence intensity (bottom) for each histogram.

The above results indicated that HIV-1 replication and release were restricted in IFNα and LPS-induced mature DCs relative to iDCs, which might be attributed to the induction of HIV-1 restriction factors in mature DCs, such as tetherin. We have reported that pro-inflammatory stimuli (such as LPS) induce DC maturation and modulate the efficiency of DC-mediated HIV-1 transmission [6]. To examine whether pro-inflammatory stimuli upregulate tetherin expression in DCs, DCs from different donors were treated with IFNα and LPS and analyzed for tetherin expression on the surface and in whole cell lysates by flow cytometry and immunoblotting, respectively. Cell surface tetherin in iDCs was low or undetectable (Figure 3A, donor 1 and 2, respectively), which correlated well with the levels of tetherin detected in whole cell lysates (Figure 3B). By contrast, high levels of surface tetherin were detected in mDC-LPS (Figure 3A), which correlated well with high levels of tetherin observed in whole cell lysates (Figure 3B). Although the surface tetherin was low or undetectable in mDC-IFNα, indicating donor variation of tetherin expression in DCs (Figure 3A), high levels of tetherin were detected in whole cell lysates (Figure 3B), suggesting that the tetherin localization in mDC-IFNα is mainly intracellular.

To examine whether other pro-inflammatory factors could induce tetherin expression, iDCs were treated with tumor necrosis factor alpha (TNF-α), which has been shown to potently induce DC maturation in our previous study [6]. TNF-α treatment of MDDCs modestly upregulated tetherin expression (Figure 3C). The specificity of the tetherin antibody was confirmed using tetherin-negative 293T cells and tetherin-positive HeLa cells (Figure 3D). Thus, treatment of DCs with pro-inflammatory stimuli causes upregulation of tetherin, but sub-cellular localization of tetherin can be dependent upon the type of stimulus.

### HIV-1 co-localizes with tetherin in mature DCs

Tetherin can show variable sub-cellular localization [27,28,42,54,55] and the localization of tetherin within a cell is critical for its antiviral function [54]. To examine the localization of HIV-1 with tetherin in mature DCs, confocal microscopy was used after a 2 h HIV-1 infection of DCs. GFP-Vpr-tagged replication-competent HIV-1 (HIV-GFP-Vpr) [56] was used to visualize the localization of HIV-1 in mature DCs. Previous studies have shown that in mDC-LPS, HIV-1 strongly concentrates in an intense patch [10] and co-localizes with the tetraspanin CD81 [12,57,58], but not with lysosomal associated membrane protein-1 (LAMP-1) [58]. Therefore, DCs were stained for CD81, LAMP-1, and tetherin to determine the sub-cellular localization of HIV-1.

**Figure 4:**
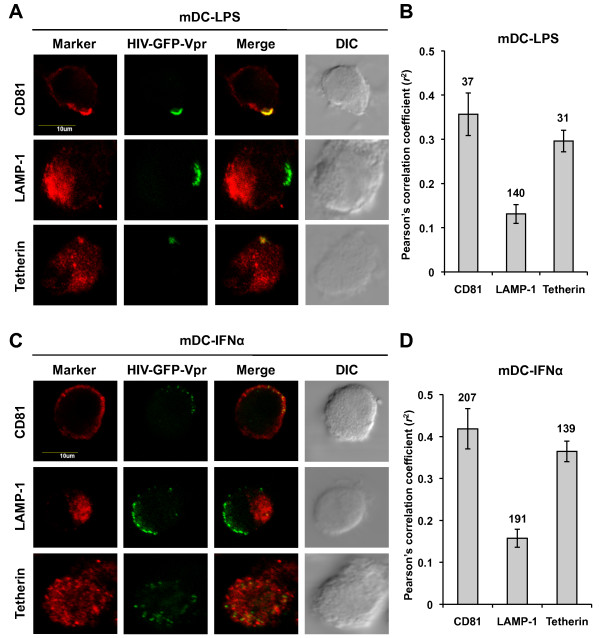
**HIV-1 localizes with CD81 and tetherin in mature DCs**. Localization of HIV-1 with cellular markers within mature DCs was assessed by confocal microscopy. (A) Representative confocal images of localization characteristics of HIV-GFP-Vpr in mDC-LPS; HIV-GFP-Vpr co-localizes with CD81 and tetherin, but not LAMP-1 in mDC-LPS. (B) Pearson's correlation coefficient analysis of mDC-LPS images. (C) Representative confocal images of localization characteristics of HIV-GFP-Vpr in mDC-IFNα; HIV-GFP-Vpr co-localizes with CD81 and tetherin, but not LAMP-1 in mDC- IFNα. (D) Pearson's correlation coefficient analysis of mDC-IFNα images. Numbers on graphic bars indicate the number of cells analyzed. Data presented are the mean ± SEM. Scale bars are 10 μm.

Consistent with previous reports [12,57,58], HIV-GFP-Vpr localized into an intense patch with CD81 and did not co-localize with LAMP-1 in mDC-LPS (Figure 4A), which was confirmed by the correlation analysis of co-localization (Figure 4B). Furthermore, the intense patch of HIV-1 observed in mDC-LPS co-localized with tetherin (Figure 4A) and the correlation analysis confirmed the co-localization (Figure 4B). In mDC-IFNα, HIV-GFP-Vpr localized into smaller patches near the plasma membrane (Figure 4C) and showed co-localization with CD81 (Figure 4C), with the correlation coefficient being similar to that observed in mDC-LPS (Figure 4B and 4D). HIV-Vpr-GFP did not co-localize with LAMP-1 in mDC-IFNα (Figure 4C) and the correlation coefficient was very low (Figure 4D). The punctate patches of HIV-1 in mDC-IFNα appeared to localize with tetherin (Figure 4C and 4D). These data indicate that in mDC-IFNα and mDC-LPS, HIV-1 localizes into distinct patches that co-localize with CD81 and tetherin but not with LAMP-1. These results suggest that LPS- and IFNα-induced tetherin expression may affect HIV-1 trafficking and transmission in mature DCs.

### Tetherin knockdown in mature DCs modestly enhances HIV-1 transmission to CD4^+ ^T cells

**Figure 5:**
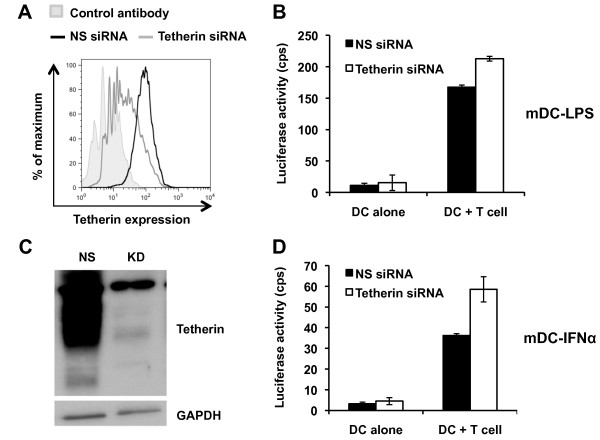
**Tetherin knockdown in mature DCs modestly enhances HIV-1 transmission to CD4^+ ^T cells**. Due to the differential localization of tetherin in matured DCs (Figure 3), tetherin knockdown was verified in (A) mDC-LPS by flow cytometry and in (C) mDC-IFNα by immunoblotting. Each plot is representative of three independent experiments performed. NS, non-silencing scramble siRNA control; KD, knockdown using tetherin siRNA. Tetherin knockdown in (B) mDC-LPS and (D) mDC-IFNα significantly enhanced transmission of single-cycle luciferase HIV-1 to Hut/CCR5 cells. Each graph represents mean results ± SEM of two independent experiments performed on DCs from different donors.

To examine the role of tetherin in mature DC-mediated HIV-1 transmission to CD4^+ ^T cells, tetherin expression in mature DCs was silenced with specific siRNA. To achieve efficient knockdown, iDCs were nucleofected with tetherin-specific or control siRNA and matured with LPS or IFNα. Analyses of tetherin expression at 2 days post nucleofection confirmed efficient knockdown of surface tetherin in mDC-LPS (Figure 5A) and total tetherin in mDC-IFNα (Figure 5C). To assess DC-mediated HIV-1 transmission, tetherin-silenced DCs were pulsed with the single-cycle luciferase reporter HIV-1 and co-cultured with the target Hut/CCR5 cells. Tetherin-silenced mDC-LPS and mDC-IFNα showed a modest 30-50% increase over the scramble siRNA controls in transmission of HIV-1 to Hut/CCR5 cells (Figure 5B and 5D), though the differences were statistically significant (*P *< 0.01). These data suggest that high levels of tetherin induced in mature DCs can modestly impair DC-mediated transmission of HIV-1 to CD4^+ ^T cells.

### Induced tetherin in mature DCs has different effects on WT and Vpu-deleted HIV-1 replication and DC-mediated HIV-1 transmission to CD4^+ ^T cells

**Figure 6:**
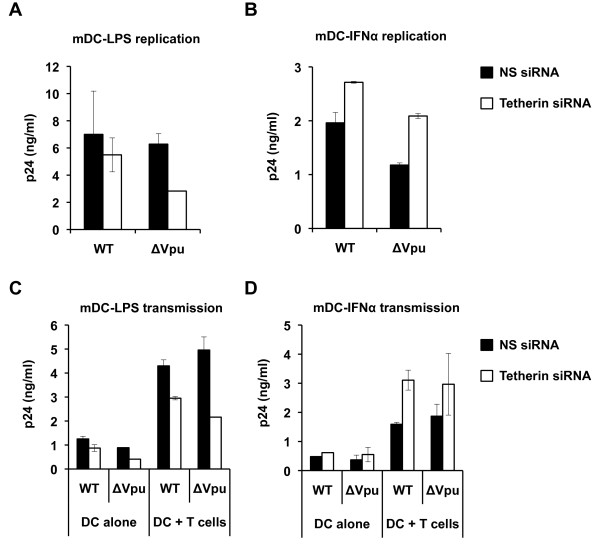
**Induced tetherin in mature DCs has different effects on WT and Vpu-deleted HIV-1 replication and DC-mediated HIV-1 transmission to CD4^+ ^T cells**. (A and B) The effect of tetherin on HIV-1 replication in mature DCs was assessed by tetherin knockdown and infection with WT NL(AD8) or NL(AD8)ΔVpu. Supernatant p24 in mDC-LPS (A) or mDC-IFNα (B) nucleofected with tetherin-specific siRNA or a non-silencing (NS) scramble siRNA control were assessed by p24 ELISA at 5 days post-infection. (C and D) The effect of tetherin on cell-to-cell transmission of WT NL(AD8) or NL(AD8)ΔVpu from tetherin-specific or NS siRNA nucleofected mDC-LPS (C) or mDC-IFNα (D) to Hut/CCR5 cells. Supernatants were collected after 2 days of co-culture and p24 concentration was assessed by ELISA. Graphs represent data from one donor representative of at least two experiments performed on DCs from different donors. Data are presented as mean ± SEM of duplicate samples.

To further examine the role of induced tetherin in replication-competent HIV-1 infection and transmission mediated by DCs, we assessed the effect of tetherin knockdown on the release of WT and Vpu-deleted (ΔVpu) HIV-1 from infected mature DCs and on DC-mediated HIV-1 transmission to Hut/CCR5 cells. Efficient tetherin knockdown was achieved in mDC-LPS and mDC-IFNα (Figure 5A,C and data not shown). Tetherin-silenced mature DCs were infected with WT NL(AD8) or ΔVpu NL(AD8) and HIV-1 p24 in the supernatants was assessed at 5 dpi, which was generally the peak of HIV-1 replication in iDCs (Figure 2D). Tetherin knockdown in mDC-LPS had no significant effect on the release of WT HIV-1, while the release of ΔVpu HIV-1 was inhibited 2-fold upon tetherin knockdown (Figure 6A). By contrast, the release of WT and ΔVpu HIV-1 from mDC-IFNα was enhanced by 38% and 2-fold upon tetherin knockdown, respectively (Figure 6B). HIV-1 infections of tetherin-silenced mature DCs were performed three times with different donors' cells and there was no statistically significant difference in WT HIV-1 release. Thus, induced tetherin expression in mature DCs does not play a major role in restriction of WT HIV-1 replication.

We next assessed the effect of tetherin knockdown on WT and ΔVpu HIV-1 transmission from mature DCs to CD4^+ ^T cells. Upon tetherin knockdown in mDC-LPS, transmission of WT and ΔVpu HIV-1 was inhibited by 25% and 2-fold, respectively (Figure 6C). By contrast, upon tetherin knockdown in mDC-IFNα, transmission of WT HIV-1 was enhanced 2-fold, while transmission of ΔVpu HIV-1 was not significantly affected (Figure 6D). As a background control of HIV-1 transmission assays, there were low levels of HIV-1 release from HIV-1-infected DC alone samples (Figure 6C and 6D). Together, these results suggest that induced tetherin in mDC-LPS and mDC-IFNα has different effects on ΔVpu HIV-1 replication and transmission, which might be due to the distinct tetherin localization in these cells.

### HIV-1 replication in iDCs upregulates tetherin independently of Vpu

**Figure 7:**
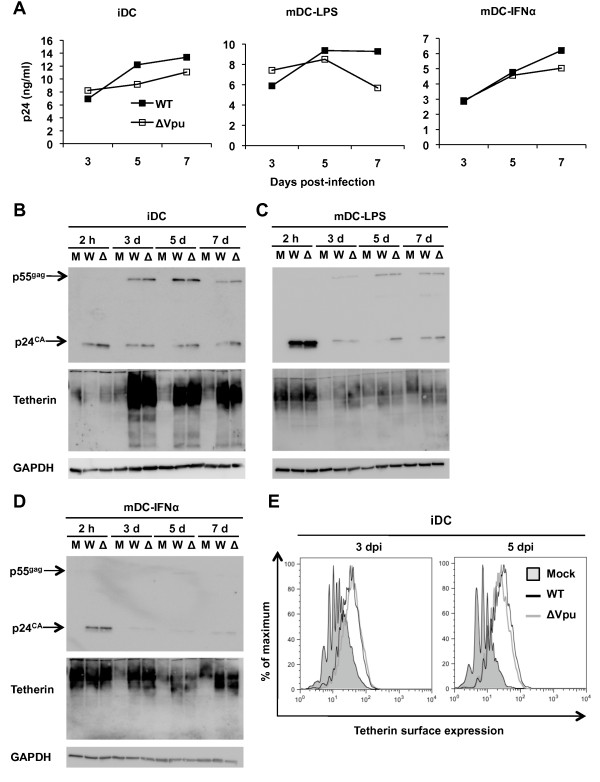
**HIV-1 replication in iDCs upregulates tetherin independently of Vpu**. (A) Supernatants from DCs infected with WT HIV-1 NL(AD8) or NL(AD8)ΔVpu were assessed for p24 concentration to quantify HIV-1 release. Cell lysates from iDC (B), mDC-LPS (C), and mDC-IFNα (D) infected with HIV-1 or mock infected were detected by immunoblotting for the expression of HIV-1 Gag (p55 and p24), tetherin, and GAPDH at 2 h, 3, 5 and 7 days post-infection (dpi). M, mock infection; W, WT NL(AD8); Δ, NL(AD8)ΔVpu. (E) Flow cytometry analyses of cell surface tetherin expression in iDCs infected with WT NL(AD8), NL(AD8)ΔVpu or mock infected at 3 and 5 dpi. Similar results have been observed in at least three independent experiments using DCs from different donors.

To examine the role of Vpu and tetherin interactions in HIV-1 infection of DCs, DCs were separately infected with WT NL(AD8) and ΔVpu HIV-1, and viral replication was assessed by p24 production in the supernatants over a time course. There was no significant defect in p24 production from infected iDCs and mDC-IFNα when Vpu was absent (Figure 7A). A 40% decrease of p24 release was observed from mDC-LPS at 7 dpi in the absence of Vpu (Figure 7A), suggesting that Vpu could partially counteract tetherin-mediated restriction of HIV-1 release.

HIV-1 infection of certain cell types can modulate tetherin surface expression [28,59,60]. However, no study has examined the effect of HIV-1 infection on tetherin expression in DCs. To assess whether HIV-1 infection affects the level of tetherin expression in DCs, iDCs, mDC-LPS and mDC-IFNα were separately infected with WT NL(AD8) and ΔVpu, and the expression of tetherin and HIV-1 Gag in DCs at 2 h and 3-7 days post-infection were assessed by immunoblotting. The p24 bands detected in all DC types at 2 h post-infection were from input HIV-1 associated with DCs (Figure 7B-D), and mDC-LPS efficiently endocytosed HIV-1 (Figure 7C). In iDCs infected with WT and ΔVpu HIV-1, there was a clear emergence of Gag p55 and p24, indicative of virus replication, and there was a corresponding induction of tetherin expression at 3 dpi (Figure 7B). Tetherin expression in HIV-1 infected iDCs appeared to diminish over time in a Vpu-independent manner (Figure 7B). These results suggest that HIV-1 infection of iDCs induces significant tetherin expression despite Vpu expression. In mDC-LPS and mDC-IFNα, high levels of DC maturation-induced tetherin were detected at 2 h post-infection, but the levels of tetherin in the mock-infected controls diminished after 3 dpi (Figure 7C and 7D). HIV-1-infected mature DCs showed consistently higher tetherin expression than mock infected controls, which also diminished over time in a Vpu-independent manner (Figure 7C and 7D). Notably, in mDC-IFNα, when low levels of HIV-1 productive replication were observed at 7 dpi (Figure 2D and 7D), there was a slight increase in tetherin expression (Figure 7D), suggesting that HIV-1 replication can induce tetherin expression in DCs. Furthermore, we compared cell surface levels of tetherin expression in WT and ΔVpu HIV-1 infected iDCs. Flow cytometry analysis confirmed that WT HIV-1 and ΔVpu-infected iDC similarly upregulated tetherin surface expression at 3 and 5 dpi compared with mock-infected cells (Figure 7E).

### HIV-1 replication in iDCs upregulates tetherin in a Nef-dependent manner

**Figure 8:**
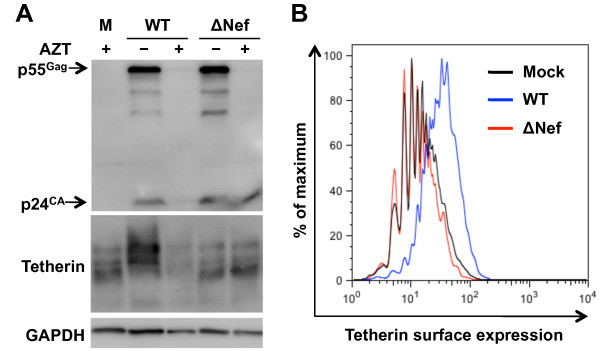
**HIV-1 replication in iDCs upregulates tetherin in a Nef-dependent manner**. (A) Cell lysates from iDCs infected with WT NL(AD8), NL(AD8)ΔNef with (+) or without (-) AZT or mock infected were evaluated at 5 dpi for the expression of HIV-1 Gag (p55 and p24), tetherin and GAPDH by immunoblotting. (B) Flow cytometry analysis of surface tetherin expression in infected DCs at 5 dpi. M, Mock infection; WT, wild-type NL(AD8); ΔNef, NL(AD8)ΔNef. One representative experiment out of three is shown.

A previous study suggested that the upregulation of tetherin surface expression by HIV-1 infection in macrophages appears to be Nef-dependent [59]. To investigate whether tetherin induction by HIV-1 in DCs was dependent on Nef synthesized during viral infection, iDCs were separately infected with WT NL(AD8) and Nef-deleted mutant (ΔNef) in the presence or absence of AZT. The expression of tetherin and HIV-1 Gag in DCs was assessed by immunoblotting at 5 dpi, which represented the peak of HIV-1 replication in iDCs (Figure 2D). WT HIV-1 infection of iDCs induced tetherin expression at 5 dpi, which could be abolished by AZT treatment (Figure 8A). The ΔNef HIV-1 mutant failed to induce tetherin, despite similar Gag production relative to WT HIV-1 infection (Figure 8A). Furthermore, flow cytometry analysis of tetherin expression in infected DCs confirmed that WT HIV-1 but not ΔNef mutant induced tetherin surface expression (Figure 8B). Thus, HIV-1 replication in iDCs induces transient upregulation of tetherin expression due to the production of Nef.

### Nef enhances the expression levels of mRNA encoding IFN-induced protein with tetratricopeptide repeats 1 (IFIT-1) in HIV-1-infected iDCs

**Figure 9:**
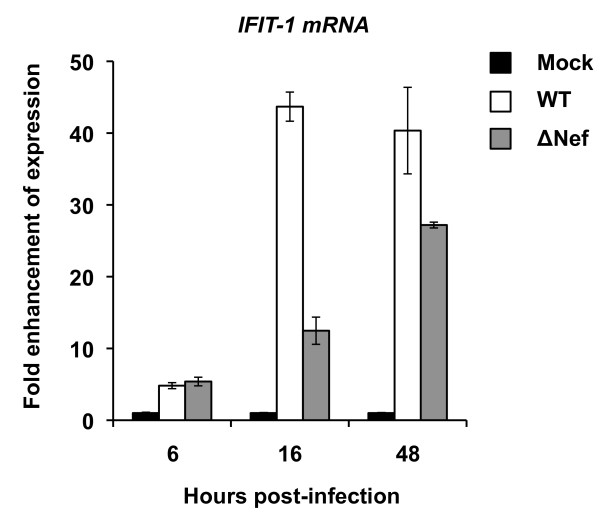
**Nef enhances the expression levels of *IFIT-1 *mRNA in HIV-1-infected iDCs**. The levels of *IFIT-1 *mRNA in iDCs mock infected or infected with WT NL(AD8) or NL(AD8)ΔNef were quantified using real-time RT-PCR. The *IFIT-1 *expression levels were normalized by the relative *GAPDH *expression levels. The results of mock-infected controls were assigned as 1 and the fold enhancement of *IFIT-1 *expression in HIV-1 infected iDCs is shown. WT, wild-type NL(AD8); ΔNef, NL(AD8)ΔNef. One representative experiment out of three is presented.

To explore the underlying mechanisms of Nef-dependent tetherin induction in HIV-1-infected iDCs, we quantified mRNA levels of *IFIT-1*, an IFN stimulated gene (ISG), in WT HIV-1 NL(AD8) or ΔNef-infected iDCs using real-time RT-PCR. Compared with mock infection, WT and ΔNef HIV-1 infections in iDCs resulted in a 5-fold increase of *IFIT1 *mRNA levels at 6 hr post-infection (Figure 9). At 16 and 48 hr post-infection, WT HIV-1 increased *IFIT1 *mRNA levels in infected iDCs 44- and 40-fold, respectively, relative to mock infection. By contrast, ΔNef HIV-1 infection in iDCs increased *IFIT1 *mRNA levels 13- and 27-fold at 16 and 48 hr post-infection, respectively, compared with mock infection (Figure 9). These data suggest that HIV-1 infection of iDCs induces ISG mRNA expression as an innate immune response, and that Nef plays an important role in this process.

## Discussion

Previous studies indicated that IFNα treatment partially inhibits post-entry HIV-1 replication and cell-to-cell transmission in CD4^+ ^T cells and macrophages [18-20], suggesting type I IFN-mediated anti-HIV responses. Compared with iDCs, mDC-LPS do not support productive HIV-1 replication, but mediate highly efficient cell-to-cell transmission [2,6,9,11]. To better understand the innate immune response of DCs to HIV-1 infection, we investigated the effect of IFNα on HIV-1 replication and cell-to-cell transmission using primary human DCs. We found that DC-mediated HIV-1 transmission and viral replication were impaired in mDC-IFNα. IFNα induced DC maturation but did not affect overall expression levels of HIV-1 receptors and the attachment factor DC-SIGN, suggesting that mDC-IFNα can mediate HIV-1 binding and entry. Compared with mDC-LPS, mDC-IFNα-mediated transmission of HIV-1 to CD4^+ ^T cells was significantly lower. Our data suggest that IFNα treatment of DCs induces an antiviral response to block HIV-1 replication and cell-to-cell transmission.

IFNα is a major inducer of tetherin expression [27,61], and other pathogenic stimuli have been suggested to stimulate tetherin expression as part of the innate immune response [32]. We thus evaluated any link between tetherin and the inhibition of HIV-1 replication and cell-to-cell transmission in DCs by assessing the levels of tetherin expression in iDCs and mature DCs. We observed that iDCs were extremely low or negative for tetherin expression, while mDC-LPS showed high levels of tetherin expression at the cell membrane. By contrast, mDC-IFNα were negative, or expressed very low levels of surface tetherin despite high levels of whole cell tetherin expression, indicating that IFNα-induced tetherin is mainly confined to the intracellular compartment in mDC-IFNα. Given the different expression levels and apparent sub-cellular localization patterns of tetherin within DCs, we therefore investigated any link between the high levels of tetherin induced in mature DCs and the HIV-1 replication and cell-to-cell transmission phenotypes observed in these cells.

Tetherin localization and co-localization with HIV-1 is vital to its restriction function, as it must tether the newly formed HIV-1 virions to the cell membrane [37,49,54,55,61]. The localization of HIV-1 in mDC-IFNα may contribute to restricted HIV-1 transmission to CD4^+ ^T cells. HIV-1 and CD81 strongly co-localized in mDC-LPS, with clear evidence of concentration of CD81 at the site of HIV-1 binding, as expected [12,57,58]. Co-localization of HIV-1 and CD81 was also observed in mDC-IFNα, but there was no evidence of a concentration of CD81 at the sites of HIV-1 binding, suggesting that the localization of HIV-1 is distinct from that observed in mDC-LPS. The lack of co-localization between HIV-1 and LAMP-1 in mDC-LPS and mDC-IFNα indicates that HIV-1 did not traffic to the lysosome for degradation in either cell type at 2 h post-infection. In both mDC-LPS and mDC-IFNα, co-localization of tetherin and HIV-GFP-Vpr was observed, which raises questions about whether tetherin affects incoming HIV-1 captured by mature DCs.

We sought to investigate the direct effect of tetherin on the replication and DC-mediated transmission of HIV-1 by silencing tetherin in mDC-LPS and mDC-IFNα. Recent studies of the tetherin function in HIV-1 cell-to-cell spread have focused on viral transmission from infected CD4^+ ^T cells to uninfected cells [48-50]. However, in DC-mediated HIV-1 transmission, DC-captured virus is concentrated at, or near, the cell surface and can be transmitted to CD4^+ ^T cells without productive replication in DCs [2,6,9-12,62]. Furthermore, the major role of tetherin is to prevent the release of fully formed HIV-1 virions from the cell surface [27,37,55], rather than affecting incoming virions. In a single-cycle HIV-1 transmission assay, tetherin knockdown in mDC-LPS and mDC-IFNα resulted in a modest increase of viral transmission to CD4^+ ^T cells, suggesting that high levels of induced tetherin in mature DCs may partially impair DC-mediated transmission of incoming HIV-1 to CD4^+ ^T cells. It is possible that tetherin siRNA may have potential off-target effect, which should be considered in transient tetherin downregulation in primary DCs. Recent studies indicated that tetherin knockdown in CD4^+ ^T cells reduces the formation of the virological synapse and HIV-1 cell-to-cell transmission [48,49]. Whether tetherin knockdown affects the formation of the virological synapse between DCs and CD4^+ ^T cells remains to be established.

A recent study indicated that tetherin expressed on target Sup-T1 cells can promote HIV-1 cell-cell transmission [50]. In this study, we focused on the role of tetherin in donor DCs and used Hut/CCR5 T cells as targets in DC-mediated HIV-1 transmission assays. Hut/CCR5 cells express high levels of endogenous tetherin (data not shown), while primary human CD4^+ ^T cells express variable levels of tetherin [49,63]. Using Hut/CCR5 cells avoided donor variations of tetherin expression in primary CD4^+ ^T cells. However, it remains to be investigated whether tetherin expressed in CD4^+ ^T cell targets affects DC-mediated HIV-1 transfer.

Tetherin inhibits HIV-1 release from cells, and its function is antagonized by Vpu [27,37]. We investigated HIV-1 replication and release in mature DCs using tetherin knockdown and a Vpu-defective mutant. The effect of tetherin knockdown on HIV-1 release from mature DCs appears to be dependent upon the maturation stimulus used and on the expression of Vpu by the virus. Our data demonstrate that tetherin expression alone is not responsible for restriction of WT HIV-1 replication in mature DCs. Indeed, previous work has identified other mechanisms responsible for post-entry restriction of HIV-1 replication in mDC-LPS [6]. The restriction of HIV-1 replication in mDC-IFNα may be due to multiple restriction factors. For example, APOBEC3G can block HIV-1 infection in DCs, and its expression is upregulated by IFNα and LPS [24,64].

In iDCs, which do not express high levels of endogenous tetherin, there was a significant increase in tetherin expression in response to the infection with WT and ΔVpu HIV-1. This is consistent with earlier studies that endogenous tetherin in macrophages can be upregulated by HIV-1 infection [59]. When mDC-LPS and mDC-IFNα were infected with WT or ΔVpu HIV-1, tetherin expression was maintained longer than that in mock-infected controls, which is presumably due to stabilization of tetherin or replenishment by tetherin induction. The maintenance of tetherin expression within mature DCs does not appear to be affected by Vpu expression.

Upregulation of surface tetherin in macrophages by HIV-1 infection appears to be induced by Nef [59], and HIV-1 replication in a human CD4^+ ^cell line causes tetherin induction after an initial down-modulation of tetherin [28]. Thus, we investigated the role of both HIV-1 replication and Nef protein in the transient induction of tetherin in iDCs. AZT treatment and deletion of Nef blocked the HIV-1-mediated tetherin upregulation in iDCs. These data suggest a role of newly synthesized Nef in the transient upregulation of tetherin in iDCs. HIV-1 Nef can cause induction of pro-inflammatory cytokines from human DCs and macrophages [65,66], so it is possible that these cytokines act in an autocrine manner to induce transient tetherin expression in DCs as part of an innate immune response to HIV-1 infection. Moreover, an increase in cellular content of tetherin may reflect its stabilization or a slow turn-over upon HIV-1 infection and expression of Nef. The mechanisms by which Nef induces tetherin expression in DCs remain to be elucidated.

Of note, a recent study indicated that HIV-1 infection of MDDCs undermines the IFN induction pathway *via *interferon regulatory factor 1 (IRF1) and blocks type I IFN production, although HIV-1 infection in DCs induces a subset of ISGs [67]. In agreement with this report, we were not able to detect the release of IFNα or IFNβ in the supernatants from HIV-1-infected iDCs at 1 to 5 dpi despite significant increases of *IFIT-1 *mRNA expression.

It is unclear as to why HIV-1 has not evolved a mechanism to block Nef-dependent induction of tetherin in DCs in addition to expressing Vpu as an antagonist of tetherin. Given the apparent transient nature of the Nef-induced tetherin expression in DCs, it is possible that as the tetherin level naturally diminishes over time, it does not affect HIV-1 release at time points of significance. HIV-1-induced tetherin expression also has the potential to ensure that HIV-1 remains in close association with the cell. In the case of DCs, this may allow HIV-1 to stay in close association with the cells during trafficking to the lymph node and subsequent transmission to CD4^+ ^T cells at late time points, as is suggested to occur *in vivo *[2,5].

In summary, we have investigated the role of IFNα and tetherin in DC-mediated HIV-1 infection and transmission. Our data suggest that tetherin is not a major restriction factor for WT HIV-1 replication in DCs or DC-mediated cell-to-cell transmission of HIV-1 to CD4^+ ^T cells. Interestingly, we found that HIV-1 infection of iDCs induces Nef-dependent tetherin expression, suggesting an intrinsic antiviral mechanism in DCs triggered by productive HIV-1 infection and the pathogenic factor Nef. Further studies of this mechanism in DCs will provide a better understanding of the innate immune response against HIV-1 infection.

## Conclusions

The restriction of HIV-1 replication and transmission in IFNα-induced mature DCs indicates a potent anti-HIV-1 response; however, high levels of tetherin induced in mature DCs cannot significantly restrict WT HIV-1 release and DC-mediated HIV-1 transmission. Nef-dependent tetherin induction in HIV-1-infected immature DCs suggests an innate immune response of DCs to HIV-1 infection.

## Methods

### Plasmids and HIV-1 stocks

Single-cycle luciferase reporter HIV-1 was generated by co-transfection of HEK293T cells with pLai3ΔenvLuc2 (a kind gift from Michael Emerman) and an expression plasmid for R5-tropic HIV-1_JRFL _envelope protein as described [68]. The infectivity of viral stocks was assessed by limiting dilution in GHOST/R5 cells as described [69]. R5-tropic, replication-competent HIV-1 strain NL(AD8) and its derivates, NL(AD8)ΔVpu, and NL(AD8)ΔNef were generated by transfection of HEK293T cells separately with pNL(AD8) [70] (a kind gift from Eric Freed), pNL(AD8)ΔVpu [71] (a kind gift from Klaus Strebel) or pNL(AD8)ΔNef [72] (a kind gift from Olivier Schwartz) as described [68]. HIV-GFP-Vpr was generated by co-transfection of HEK293T cells with pNL(AD8) and a Vpr-GFP expression vector pGFP-Vpr (a kind gift from David McDonald) as described [56]. Gag p24 concentrations of viral stocks were measured using an enzyme-linked immunosorbent assay (ELISA; The AIDS Vaccine Program, SAIC, Frederick, MD).

### Cell culture

Monocytes were isolated from buffy coats (American Red Cross Blood Service, Columbus, Ohio) by Histopaque and Percoll (Sigma-Aldrich) gradient centrifugation as described [68]. All DCs utilized in this study were in culture for 7 days post-monocyte isolation. iDCs were generated by incubation of the monocytes in the presence of interleukin 4 and granulocyte-macrophage colony stimulating factor for 7 days as described [68]. LPS-induced mature DCs (mDC-LPS) were generated by addition of 10 ng/ml LPS (*Escherichia coli *strain O55:B5; Sigma-Aldrich) to day 5 iDCs and subsequently cultured for an additional 2 days. IFN-α matured DCs (mDC-IFN-α) were generated by addition of 2,000 IU/ml of recombinant IFN-α-A/D (Sigma-Aldrich) to day 6 iDCs and subsequently cultured for an additional one day. iDCs were treated with TNF-α (PeproTech) at the indicated concentrations and cultured for 24 hr before immunoblotting analysis of tetherin expression. HEK293T and HeLa cell lines were maintained in DMEM supplemented with 10% fetal calf serum, L-glutamine and penicillin and streptomycin. Hut/CCR5 cells (kind gift from Vineet KewalRamani) were grown in selective media as previously described [52].

### Flow cytometry analysis of surface marker expression

DCs were stained with phycoerythrin (PE)- or fluorescein isothiocyanate (FITC)-conjugated antibodies to CD4 (clone number S3.5; Invitrogen), DC-specific intercellular adhesion molecule-3-grabbing non-integrin (DC-SIGN, clone number 120507; R&D Systems) or CD86 (clone number BU63; Invitrogen). Negative controls were antibodies matched for isotype and fluorescent conjugates: mouse IgG_2a _(PE conjugate; BD Biosciences), mouse IgG_2 _(FITC conjugate; BD Biosciences) or mouse IgG_1 _(FITC conjugate; Invitrogen). For CCR5 staining, DCs were stained with a purified antibody to CCR5 (clone number 3A9; BD Biosciences), followed by a FITC-conjugated goat anti-mouse secondary antibody (Caltag) as described [53]. Cells were stained for surface tetherin expression using a rabbit serum against tetherin [55] in conjunction with a PE-conjugated goat anti-rabbit F_ab _fragment (BD Biosciences). Stained cells were analyzed on a Guava EasyCyte Mini (Millipore) flow cytometer and data were processed using the FlowJo software (Tree Star). Statistics for percentage positive cells were established by setting up a histogram gate equivalent to 1% on the relevant negative control cells and utilizing the same gate on stained cells.

### Immunoblotting

Cell lysates were prepared using a Cell Lysis Buffer (Cell Signaling) supplemented with the Protease Inhibitor Cocktail (Sigma-Aldrich) according to the manufacturer's instructions. Following protein quantification using a BCA kit (Pierce), 10 μg of each lysate was run on an 8%:13% SDS-polyacrylamide gel or a 12.5% Criterion pre-cast gel (Bio-Rad) and transferred to polyvinylidine fluoride membrane (Millipore) using an Electrophoretic Transfer Cell or Semi-Dry Elecrophoretic Transfer Cell (Bio-Rad). Blots were stained with either the anti-tetherin rabbit serum [55], an anti-HIV-1 p24 (clone number 24-2, the NIH AIDS Research and Reference Reagent Program), or an anti-GAPDH (Imgenex), followed by a relevant horseradish peroxidase-conjugated secondary (anti-rabbit or anti-mouse, Promega). Blots were stripped for subsequent probes using Western Blot Stripping Buffer (Thermo Scientific) according to the manufacturer's instructions. Blots were visualized using West Pico chemiluminescent substrates (Thermo Scientific) and a FujiQuick imager (FujiFilm).

### Confocal microscopy

DCs (2 × 10^5^) were pulsed with HIV-Vpr-GFP (20 ng of p24) for 2 h, then washed once with phosphate buffer saline (PBS) and prepared for confocal microscopy [57]. Cells were adhered to a poly-L-lysine coated slide, fixed and permeabilized as described [68]. Cells were labeled using antibodies to CD86 (clone number IT2.2, BD Pharmingen), LAMP-1 (clone number H4A3, BD Pharmingen), and tetherin followed by Alexi-Fluor-568 conjugated anti-mouse or anti-rabbit secondary antibodies (both from Invitrogen). Slides were sealed with Gold anti-fade reagent (Invitrogen) and analyzed on an FV100-Spectral or FV100-Filter confocal microscope (Olympus). Images were processed and co-localization analyses were performed using the FV10-ASW 2.0 Viewer (Olympus).

### siRNA knockdown of tetherin in matured DCs

It has been shown that iDCs are far more receptive to nucleofection than mDC-LPS [6]. To efficiently knockdown tetherin in mature DCs, iDCs were nucleofected with a specific siRNA pool against tetherin and subsequently matured with LPS as described [62]. Amaxa nucleofector and a DC-specific nucleofection kit (Lonza) were used to nucleofect iDCs, according to the manufacturer's instruction. iDCs (2 × 10^6^) were nucleofected with 3 μg of a nonspecific siRNA control or a specific siRNA (siGENOME SMARTPOOL) targeting BST-2 (Dharmacon). Nucleofected cells were subsequently cultured for 1 day in DC culture media, and then activated with 100 ng/ml of LPS or 2,000 U/ml of IFN-α for 1 day to generate mature DCs.

### HIV-1 infection and transmission assays

DCs (2 × 10^5^) at day 7 of culture were challenged with HIV-1 (20 ng of p24) for 2 h as described [11]. HIV-1 infected DCs were washed once with PBS to remove unbound virions and subsequently cultured for indicated times. Cell lysates and supernatants were collected at indicated times for assessment of p24 concentration by ELISA. Samples were lysed using 1% Triton X-100 for 1 h at 37°C. Where indicated, cells were treated with 1 μM of AZT (NIH AIDS Research and Reference Reagent Program) for the duration of HIV-1 infection and subsequent culture. DC-mediated HIV-1 transmission assays (4 ng of p24 equivalent HIV-1 input) were performed using the Hut/CCR5 cells as target cells as described [6].

### RT-PCR quantification of IFIT-1 expression in HIV-1-infected iDCs

iDCs (2.5 × 10^6^) were mock infected or infected with HIV-1 NLAD8 or NLAD8ΔNef for 2 hr, washed with PBS and subsequently cultured for 6, 16 and 48 hr. At each indicated time point, cells were harvested and total cellular RNA was extracted using the RNeasy Mini kit (Qiagen) and treated with RNase-free DNase (Ambion). cDNA was synthesized using the SuperScript III first-strand synthesis system (Invitrogen) according to the manufacturer's instruction. The relative levels of cDNA were assessed for spliced *glyceraldehyde-3-phosphate dehydrogenase *(*GAPDH*) using specific primers (forward, 5'-GGA AGG TGA AGG TCG GAG TCA ACG G-3'; reverse, 5'-CTG TTG TCA TAC TTC TCA TGG TTC AC-3'), and for *IFIT-1 *using specific primers (forward, 5'-CAA CCA TGA GTA CAA ATG GTG-3'; reverse, 5'-CTC ACA TTT GCT TGG TTG TC-3'). Real-time PCR was performed with the iQ SYBER Green kit (Bio-Rad) using the CFX96 real-time system as previously described [6].

### Statistical analysis

Data were analyzed using a two-way ANOVA test and Bonferroni post-test. Statistical significance was defined as *P *< 0.05.

## Competing interests

The authors declare that they have no competing interests.

## Authors' contributions

LW conceived the study, designed the experiments and participated in data analyses. CMC performed all the experiments and participated in the experimental design. PS contributed to some experiment design and data analyses and provided tetherin anti-serum. CMC and LW wrote the manuscript. All authors read and approved the final manuscript.
